# A Marine Natural Product, Harzianopyridone, as an Anti-ZIKV Agent by Targeting RNA-Dependent RNA Polymerase

**DOI:** 10.3390/molecules29050978

**Published:** 2024-02-23

**Authors:** Kexin Zhang, Jingyao Liang, Bingzhi Zhang, Lishan Huang, Jianchen Yu, Xuhan Xiao, Zhenjian He, Huaming Tao, Jie Yuan

**Affiliations:** 1 School of Public Health, Sun Yat-sen University, Guangzhou 510080, China; zhangkx39@mail2.sysu.edu.cn (K.Z.); liangjy228@mail2.sysu.edu.cn (J.L.); 2 Key Laboratory of Tropical Disease Control (Sun Yat-sen University), Ministry of Education, Guangzhou 510080, China; bingzhi0908@163.com (B.Z.); yujchen@mail2.sysu.edu.cn (J.Y.); xiaoxh26@mail2.sysu.edu.cn (X.X.); 3 School of Pharmacy, Guangdong Pharmaceutical University, Guangzhou 510006, China; 4 School of Traditional Chinese Medicine, Southern Medical University, Guangzhou 510515, China; huanglishan0527@163.com; 5 Zhongshan School of Medicine, Sun Yat-sen University, Guangzhou 510080, China

**Keywords:** harzianopyridone, ZIKV, marine natural products, antiviral drugs, RdRp

## Abstract

The Zika virus (ZIKV) is a mosquito-borne virus that already poses a danger to worldwide human health. Patients infected with ZIKV generally have mild symptoms like a low-grade fever and joint pain. However, severe symptoms can also occur, such as Guillain-Barré syndrome, neuropathy, and myelitis. Pregnant women infected with ZIKV may also cause microcephaly in newborns. To date, we still lack conventional antiviral drugs to treat ZIKV infections. Marine natural products have novel structures and diverse biological activities. They have been discovered to have antibacterial, antiviral, anticancer, and other therapeutic effects. Therefore, marine products are important resources for compounds for innovative medicines. In this study, we identified a marine natural product, harzianopyridone (HAR), that could inhibit ZIKV replication with EC_50_ values from 0.46 to 2.63 µM while not showing obvious cytotoxicity in multiple cellular models (CC_50_ > 45 µM). Further, it also reduced the expression of viral proteins and protected cells from viral infection. More importantly, we found that HAR directly bound to the ZIKV RNA-dependent RNA polymerase (RdRp) and suppressed its polymerase activity. Collectively, our findings provide HAR as an option for the development of anti-ZIKV drugs.

## 1. Introduction

Zika virus (ZIKV) is a small, enveloped positive-strand RNA virus with an icosahedral symmetrical structure of round particles about 40–70 nm in diameter [1]. It is a mosquito-borne virus belonging to the flavivirus types of the *flavivirus* family [2]. In 1947, ZIKV was first isolated from rhesus monkeys through a *flavivirus* surveillance network in the Zika Forest in Uganda, Africa [3]. For decades, ZIKV infections have been sporadic in humans [4]. The first ZIKV epidemic was reported in Micronesia, where nearly 73% of local residents were infected [5]. Subsequently, another large outbreak broke out in French Polynesia [6], which was followed by an outbreak of ZIKV infections in the Americas in 2015 [7]. Since then, ZIKV has been reported in 89 countries and districts, becoming a severe global public health challenge [8,9]. Most people infected with ZIKV are asymptomatic [10]. Even when symptoms occur, they are generally mild, such as rashes, fever, and muscle and joint pain [11]. However, ZIKV infection should not be ignored, as pregnant women infected with ZIKV may cause microcephaly in newborns [12]. Moreover, infected people can also lead to Guillain-Barré syndrome [13], neuropathy, and myelitis [14]. Currently, there is no targeted treatment for ZIKV infection or disease [15]. The recommended treatments are symptomatic management. Acetaminophen is often used clinically for fever and antihistamines for itchy rashes [2]. Thus, there is still a powerful demand for the development of anti-ZIKV drugs [16].

Natural products are materials produced by living creatures. They have a variety of medicinal characteristics and bioactivities for medicinal treatment. Seventy-five percent of the Earth’s surface area is the sea, which contains 80% of the world’s species [17]. More than 60 years have passed in the research of marine medicines. To date, due to the accomplishments of marine natural products, accomplishments have also been achieved in the development and application of clinical medication [18]. Some natural products have long been the traditional source of medication molecules. As an important resource for human natural medicine research, many metabolites produced by marine organisms not only have a unique chemical structure, but also have significant biological activity and high medicinal value. In addition, marine natural products have been indicated to have significant antibacterial, antiviral, antitumor, antioxidant, and immunomodulatory functions [19,20]. Vidarabine is an antiviral drug obtained from a natural sponge prototype product. It has been used to treat herpes simplex virus type 1 and type 2 infection diseases for many years [21]. Polymannuronate phosphate (PMP) is a promising anti-SARS-CoV-2 drug, and polymannuronate monophosphate (PMPD) is the most effective ingredient to block the interaction between spike and ACE2 [22]. Sulfated chitooligosaccharide has also been proven to exert inhibitory functions on the human immunodeficiency virus (HIV), inhibiting virus entry into host cells [10]. Moreover, many marine alkaloids have biological activity against neglected tropical diseases that are caused by pathogens like protozoan parasites and malaria [23]. Polycyclic indoles of fascaplysin and homofascaplysin, extracted from the sponge *Hyrtios* cf. *erecta,* are a class of dichloromethane/methanol extracts and have anti-protozoal activity [24]. As a neglected disease, dengue affects about 50% of the world’s population, with an estimated 100 to 400 million infections occurring annually [25]. Several compounds with anti-dengue virus (DENV) activity have been isolated from the marine environment [26]. Fucoidan is a sulfated polysaccharide isolated from *Cladosiphon okamuranus*, a natural brown seaweed in Okinawa, Japan, that shows inhibitory activity against DENV-2 [27]. Diterpenes (dolastanes and ecodolastanes) isolated from *Canistrocarpus cervicornis* show inhibitory activity against DENV-1 and DENV-3 [28]. ZIKV belongs to the *Flavivirus* family. Yuan et al. have found that cyclodepsipeptides (CDPs), extracted from the sea sponge-associated fungus *Beauveria feline*, exhibit anti-ZIKV activity [29]. In Vero cells, the dichloromethane extract of the red seaweed *Bryotamnion triquetrum* inhibits ZIKV replication [30]. Above all, marine natural products are crucial for the development of new antiviral drugs, especially for neglected diseases. In this context, mining antivirals in marine natural products is beneficial in accelerating anti-ZIKV agent exploitation.

**Figure 1:**
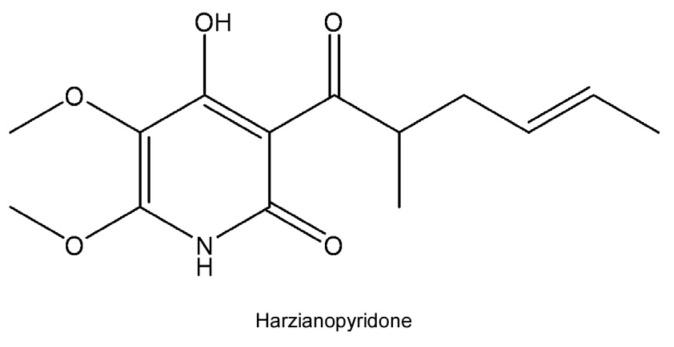
The structure of harzianopyridone (HAR).

Harzianopyridone (HAR) (Figure 1) is a volatile organic compound derived from the strain *Trichoderma* sp. That was obtained from sponge (*Callyspongia* sp.) samples. Secondary metabolites (SMs) of *Trichoderma* sp. have been proven to have antimicrobial activity against phytopathogenic fungi and are one of the possible methods used for biological prevention today. Antifungal HAR was first isolated from *Trichoderma harzianum* in 1989 [31], containing a penta-substituted pyridine ring system in a 2,3-dimethoxy-4-pyridyl pattern [32]. There are few studies on the biological activity of HAR. It has been demonstrated that HAR can effectively inhibit mitochondrial complex II [33,34]. Moreover, HAR can reduce myosin heavy chain-embryonic levels and effectively inhibit the formation of muscle cells in vitro and in vivo [35]. In our study, we found that HAR has anti-ZIKV activity in cell culture models. Subsequently, it was further clarified that the antiviral effect was manifested by direct interaction with ZIKV RNA-dependent RNA polymerase (RdRp). Above all, these results further demonstrate its possibility as a drug development.

## 2. Results

### 2.1. HAR Exerts Antiviral Potential without Obvious Cytotoxicity

**Figure 2:**
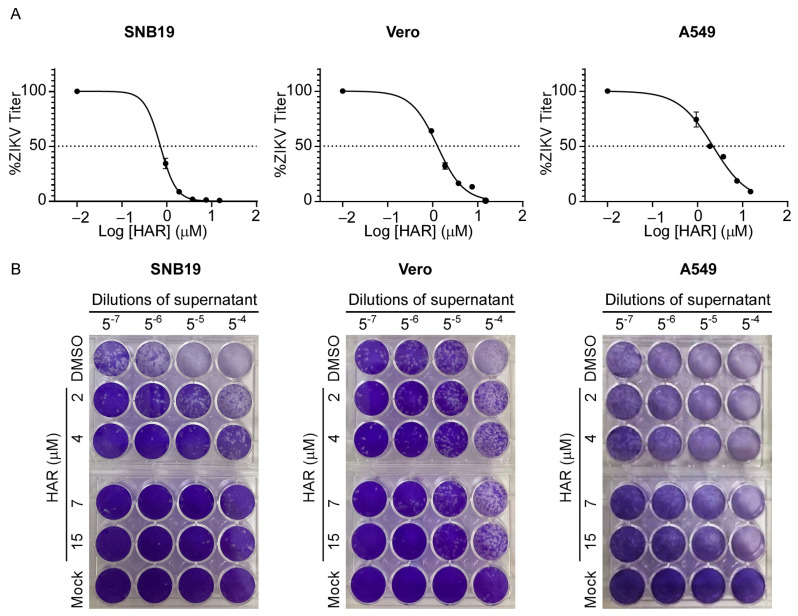
The antiviral effects of HAR in SNB19, Vero, and A549 cells. (**A**) The ZIKV titers in SNB19, Vero, and A549 cells were quantified by real-time PCR (MOI = 0.2). The supernatant was obtained from cells treated with HAR at the indicated concentrations at 48 h post-infection (hpi). (**B**) Representative images of the plaque-forming assay. On Vero cells, the plaque-forming assay was used to confirm the ZIKV titers in the supernatant. The supernatant was obtained from SNB19, Vero, and A549 cells treated with HAR at the indicated concentrations at 48 h post-infection (hpi).

To determine whether HAR could inhibit the replication of ZIKV, we infected three susceptible cell lines with ZIKV, including A549, SNB19, and Vero. Subsequently, cells were treated in medium containing different concentrations of HAR (1, 2, 4, 7, and 15 µM). The results show that HAR was effective against ZIKV in SNB19 cells, with an EC_50_ value of 0.46 ± 0.13 µM (Table 1), while showing low cytotoxicity (CC_50_ = 45.17 ± 2.16 and SI = 98.20). The results were also consistent in Vero and A549 cells, as HAR inhibited ZIKV infection with EC_50_ values from 1.40 to 2.63 µM (Table 1) and did not cause significant cell cytotoxicity (CC_50_ = 92.31 ± 6.66 in Vero cells and CC_50_ = 82.87 ± 4.94 in A549 cells). Moreover, in the above three cell lines, HAR also proved remarkably lower EC_90_ values (Table 1) compared favorably with its CC_50_ value (>45 µM). Furthermore, the plaque-forming assay also shows consistent results that HAR significantly repressed the number of live ZIKV particles (Figure 2), indicating HAR as a potent antiviral agent.

### 2.2. HAR Inhibits the Expression of ZIKV Proteins

**Figure 3:**
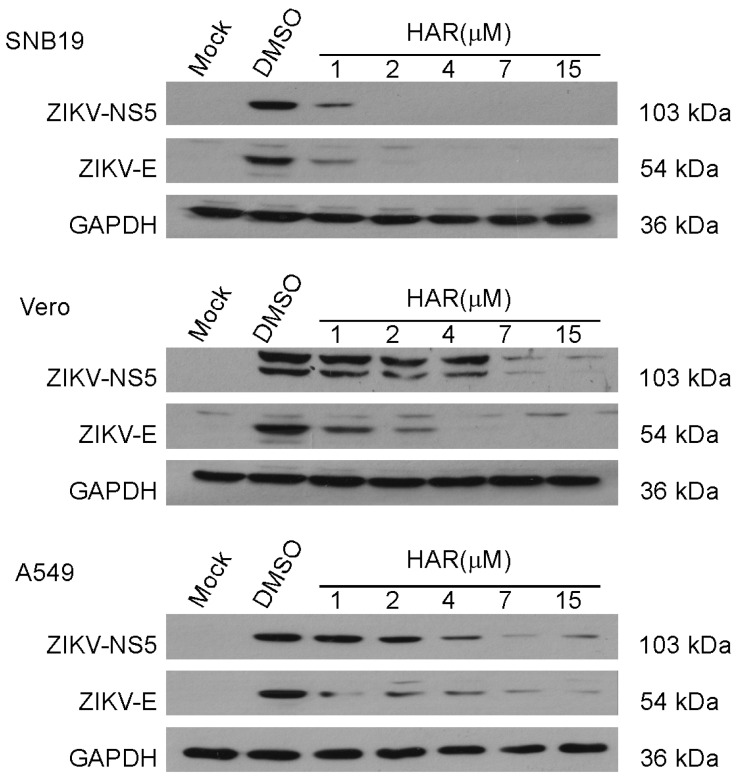
HAR inhibits the expression of ZIKV proteins. Western blotting analysis of protein expression of ZIKV NS5 and E in the cell lysates of SNB19, Vero, and A549 for the anti-ZIKV activity of HAR or DMSO at the indicated concentrations at 48 h post-infection (hpi) (MOI = 0.2).

To further clarify the antiviral effects of HAR, we determined the expression of two important ZIKV proteins in three different cell lines by Western blotting analysis. NS5 is the largest NS protein of ZIKV, which inhibits the innate immunity signal to mediate ZIKV evasion from the antiviral defense in the host [36]. By binding to a receptor with the E protein, the viruses enter the cell, making the E protein an ideal antiviral target [37]. The results show that the expression of these two important viral proteins was inhibited effectively in a dose-dependent manner after HAR treatment (Figure 3). Our results show that ZIKV proteins were not expressed in SNB19 and Vero cells when the concentration of HAR was 7 µM (Figure 3). While in A549 cells, it was 15 µM or higher concentrations of HAR that could completely inhibit the expression of ZIKV proteins (Figure 3). These results agreed with the results of the EC_50_ values against ZIKV and the plaque-forming assays in the above three cell lines.

### 2.3. HAR Protects Cells against ZIKV Infection

**Figure 4:**
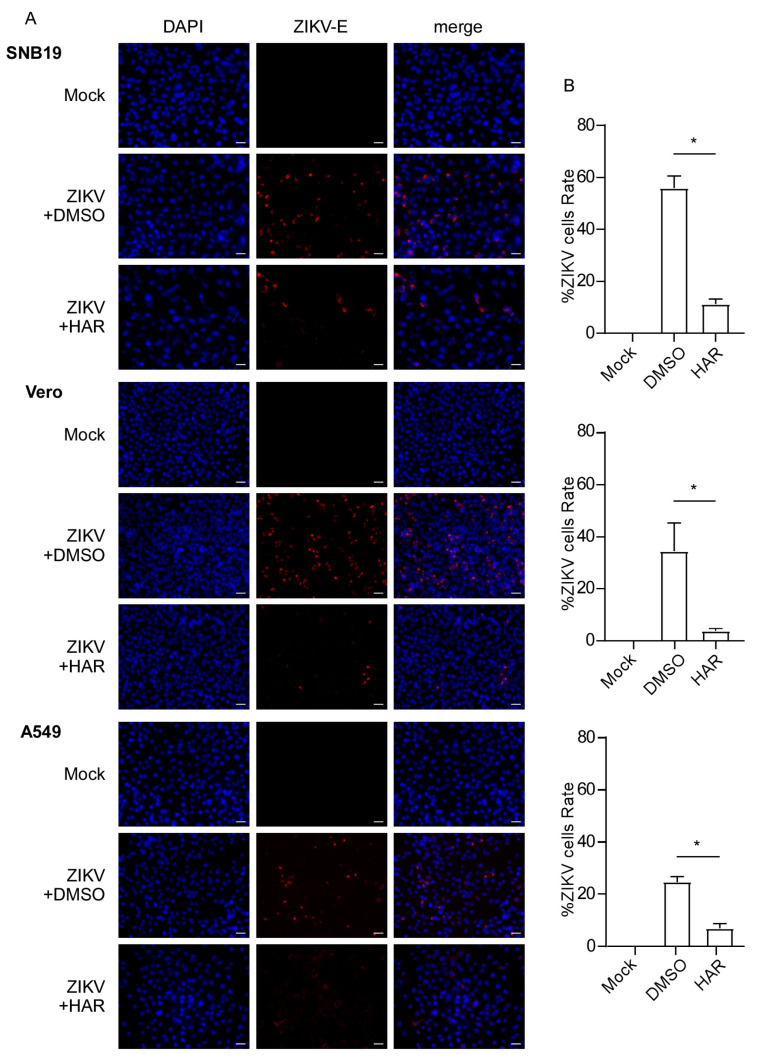
HAR protects cells against ZIKV infection. (**A**) Immunofluorescence staining for ZIKV-E in ZIKV-infected cells (MOI = 0.2) under treatment by DMSO or 7.5 µM HAR. DAPI staining (blue) is used to color the cell nuclei. ZIKV-E protein is indicated in red (scale bar, 100 µm). (**B**) Quantification of ZIKV+ cells relative to mock infection is exhibited in the histogram counted by Image J software (1.50i NIH, Bethesda, MD, USA). * *p* < 0.05.

Furthermore, we used an immunofluorescence assay to visualize the antiviral effects of HAR against ZIKV. After ZIKV infection, a large number of cells emitted red fluorescence in the control cells that were treated with DMSO (Figure 4A). While the number of cells in red fluorescence considerably decreased and even disappeared after treatment with HAR (Figure 4A), indicating that HAR protects cells against ZIKV infection, the quantitative results also indicate a huge statistical difference between the DMSO group and the HAR group (Figure 4B). These results show that HAR has great anti-ZIKV activities.

### 2.4. HAR Directly Targets the ZIKV RdRp

**Figure 5:**
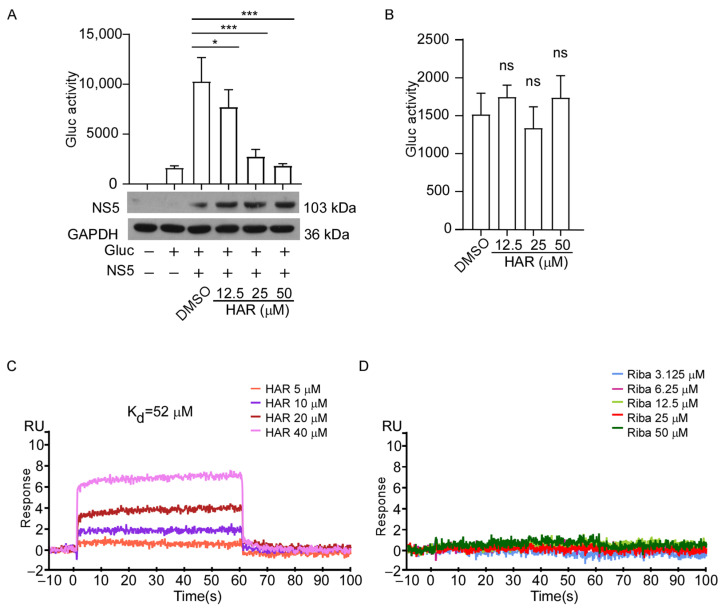
HAR directly targets the ZIKV RdRp. (**A**) HAR inhibited the activity of the ZIKV NS5 protein. 293T cells were co-transfected with the Gluc reporter and the ZIKV NS5 plasmid, as well as treated with different concentrations of HAR for 24 h. The culture medium was collected for luminescence examination. The transfection efficiency of NS5 was detected by a Western blotting assay. Used GAPDH as a loading control. * *p* < 0.05, *** *p* < 0.001. The symbol “+” means inclusion, and “−” means exclusion. (**B**) The effects of HAR on Gluc activity. 293T cells were transfected with Gluc reporter plasmid, followed by treatment with HAR (12.5, 25 or 50 µM) or 1% DMSO as a control. Determined the Gluc activities after transfection at about 24 h. “ns” indicates not significant. Experiments were performed in triplicate. (**C**,**D**) The surface plasmon resonance biosensor (SPR) assay was performed to examine the interaction between ZIKV NS5 (RdRp) protein and HAR (**C**). Ribavirin (**D**) was used as a negative control. BIAcore T100 analysis software (BIAevaluation Version 3.1) was used to analyze the K_d_ values.

We further explored the specific mechanism of the antiviral function of the HAR. ZIKV NS5 protein, which contains an RdRp domain and contributes to the replication of the viral RNA [38]. In consideration of the importance of the NS5 protein for ZIKV and the significant inhibitory effects of HAR on NS5 expression, we firstly used a mini-genome reporter system to further explore whether HAR affects NS5-mediated ZIKV RNA synthesis. The ZIKV RNA polymerase NS5 and a viral mini-genomic RNA encoding a reporter gene Gluc, in which the Gluc expression cassette was flanked by 5′ and 3′ untranslated regions (UTRs) of ZIKV, were co-transfected in 293T cells. Gluc mRNA was synthesized by cellular pol II, and then Gluc was expressed. The viral RdRp amplified the mRNA after the expression of ZIKV NS5, which led to higher Gluc levels (Figure 5A). The viral RNA synthesis by NS5 was reflected by the content of Gluc secreted into a culture medium. As shown in Figure 5A, the treatment of HAR significantly inhibited the Gluc signals, indicating impaired viral RNA synthesis. Moreover, we found that HAR treatment did not affect Gluc activity in cells expressing the reporter alone (Figure 5B). Next, we immobilized RdRp protein purified from *Escherichia coli* on a CM5 sensor chip for surface plasmon resonance biosensor (SPR)-based BIAcore experiments and investigated the direct interaction between HAR and RdRp protein. The results show that HAR could directly bind to ZIKV RdRp (K_d_ = 52 µM) (Figure 5C), while ribavirin and RdRp protein show no interaction (Figure 5D). These findings indicate that ZIKV RdRp was the target of HAR.

## 3. Discussion

In February 2016, ZIKV was declared a global public health emergency by the World Health Organization (WHO). Nowadays, ZIKV transmission has diminished, but it is still important to remain vigilant for its reemergence and global spread [4]. In addition to preventive measures, antiviral drug treatment is also an important strategy against infectious diseases [39]. To date, no specific antiviral drugs have been permitted for the treatment of ZIKV infection. Clinically, conventional drugs are often used to relieve symptoms, such as acetaminophen, which is a common treatment used to control fever and pain, and histamine antagonists, which are useful to cure itchy rashes. In this study, we identified a marine natural compound that exerted antiviral effects. In SNB19 cells, the concentration of HAR at less than 0.5 µM could inhibit half of the viral replication. In Vero and A549 cells, the EC_50_ values also did not exceed 3 µM, indicating HAR has great antiviral activities. We have previously reported a series of compounds with inhibitory activity against ZIKV. Compound **22** indicates potent anti-ZIKV activity, with an EC_50_ value ranging from 1.33 μM to 5.72 μM [40]. Dapoxetine has been found to show a concentration-dependent anti-ZIKV effect with an EC_50_ value ranging from 4.2 µM to 12.6 µM [41].Fidaxomicin inhibits half of ZIKV replication at about 10 µM in vitro [42]. In addition, amodiaquine shows inhibitory activity for ZIKV with an EC_50_ value of 3.07 µM [43]. Baz and Cai et al. demonstrated the inhibitory effect of favipiravir on multiple ZIKV strains in vitro (EC_50_ = 35 μM) [44]. There are also some marine natural products that have activity against ZIKV. The dichloromethane extract of the marine seaweed *Canistrocarpus cervicornis* exhibits an antiviral effect at 10 µM in Vero cells [45]. These data illustrate that HAR is an effective anti-ZIKV agent with an EC_50_ at low concentrations in cell culture models. Subsequently, we used a microgenomic reporter system to verify the effect of HAR on ZIKV RNA synthesis. Surface plasmon resonance (SPR) experiments demonstrate the direct binding between HAR and NS5 RdRp (K_d_ = 52 µM). Our previous reports have indicated that dapoxetine also has interaction with NS5 RdRp (K_d_ = 49.8 µM) [41] and fidaxomicin directly binds ZIKV NS5 full-length protein (K_d_ = 19 µM) [42]. All these fruitful findings are expected to provide new candidates for anti-ZIKV drug development.

ZIKV is a small enveloped positive-strand RNA virus [46]. For all RNA viruses, RdRp is a major target for medicine design, given its critical function in RNA viral replication [47,48]. For viral RdRp inhibitors, nucleoside polymerase inhibitors (NIs) and non-nucleoside polymerase inhibitors (NNIs) have been approved for viral infection. Sofosbuvir is the first clinically effective anti-HCV NI drug. The active ingredient of sofosbuvir undergoes phosphorylation and binds to the active site of RdRp in the cells. Thus, it can compete with natural substrates for RNA synthesis [49]. Favipiravir is a novel antiviral drug that characteristically and effectively inhibits RdRp in flavivirus, orthomyxovirus, norovirus, and other viruses. Nevertheless, the exact molecular mechanism of interaction with RdRp has not been fully elucidated. Evelyn J. et al. suggested that favipiravir has a lethal mutational effect on ZIKV [50]. Rilpivirine, a non-nucleoside reverse transcriptase inhibitor (NNRTI), is used to deal with HIV-1 infection. In primary human astrocytes, rilpivirine inhibits the enzymatic activity of NS5 and suppresses ZIKV infection and replication [51]. In our previous studies, we identified a series of small anti-ZIKV molecules as RdRp inhibitors, such as fidaxomicin [42], doramectin [52], and compound **22** [40], similar to HAR in this study.

The ocean is a treasure trove of natural products rich in species diversity. Marine natural products are a significant source for the research and development of original new drugs nowadays. Most of the existing marine drugs are active ingredients extracted directly from marine organisms. Some are obtained by artificial synthesis or biotechnological modification of marine biological active substances. Marine drugs are mainly divided into three categories: marine traditional Chinese drugs, marine chemical drugs, and marine biological goods. At present, the main development direction of marine drugs is to develop new drugs for major human diseases such as tumors, cardiovascular diseases, pathogenic microorganisms, and nervous system diseases [53]. Marine organisms can produce many bioactive substances and metabolites with special structures [54,55]. Marine drugs also have remarkable pharmacological stability and potency [56], relatively small toxicities and side effects [57], and have a unique effect on the prevention and treatment of cancer [58,59], AIDS [60], and cardiovascular and cerebrovascular diseases [61]. Today, marine organisms are one of the major contributors to the development of new and specialty drugs [62]. As a marine natural product, HAR is extracted from *Trichoderma* sp. that was isolated from sponges. HAR has an inhibitory effect on phytopathogenic fungi, such as *P. ultimum* and *G. graminis* var. *tritici* [63]. This is the first report that HAR could also inhibit ZIKV by suppressing viral RNA synthesis and directly targeting NS5 RdRp, increasing its biological function. In view of the drug properties, the anti-ZIKV activity of HAR in mammals still needs to be explored.

The development of new drugs is a long-term and expensive procedure with a particularly high failure rate. Repurposing “old” drugs is becoming a progressively attractive suggestion for unusual or rare diseases. It can save a lot of time and resources by significantly speeding up the drug development process. Avarone is a marine natural product obtained from the sponge *Dysidea avara* [64]. Avarone has many kinds of medicinal functions, including anti-tumor [65], antibacterial, and antiviral [66]. In recent years, it has been found that avarone can be designed as a multi-target drug. The drug can treat diabetes and its pathological complications [67]. From ZIKV and Ebola to COVID-19 in 2019, infectious diseases caused by viruses pose a significant global health burden. One of the major global medical needs is specific treatment for most viral infectious diseases. Over the past few years, drug repurposing has made a significant contribution to the identification of new antiviral molecules and therapeutic intervention targets. Remdesivir [68], chloroquine [69], and fluvoxamine [70] are considered to be promising drugs for the treatment of COVID-19. Amodiaquine, as an antimalarial drug approved by the FDA, also shows anti-ZIKV activity [43].Two of the compounds we found with anti-ZIKV activity were also repurposed by “old” drugs. One is fidaxomicin, which is a first-in-class macrocyclic antibiotic [42]; the other is dapoxetine, which can particularly inhibit serotonin reuptake [41]. Potential drugs approved for the treatment of HCV infection have also been reported, including ribavirin [71] and sofosbuvir [72], which can also inhibit ZIKV. Therefore, drug repurposing is an effective strategy for the development of antiviral drugs.

In conclusion, our results provide a potential marine natural product, HAR, against ZIKV by directly binding to the RdRp and inhibiting its polymerase activity. Based on our in vitro data, it is necessary to further verify the effectiveness and toxicity of HAR in animals. Further efforts are also needed to achieve structural optimization and transformation in the future investigation. In this study, HAR provides an alternative drug molecular backbone for the human drug molecule library, especially in anti-ZIKV drugs.

## 4. Materials and Methods

### 4.1. Cell Culture and Virus

Primary human lung adenocarcinoma cell line A549, kidney epithelial cells of African green simian Vero, and human embryonic kidney cell line 293T were obtained from the Cell Bank of the Chinese Academy of Sciences (CBCAS), Shanghai, China. Human glioblastoma cell line SNB19 (CRL-2219, ATCC, Manassas, VA, USA) was obtained from ATCC. A549, Vero, 293T, and SNB19 cells were cultured in DMEM (Invitrogen, Carlsbad, CA, USA) containing 10% fetal bovine serum (FBS) (GIBCO, Carlsbad, CA, USA), 2 mM L-glutamine, 100 μg/mL streptomycin, and 100 units/mL penicillin (Invitrogen, Carlsbad, CA, USA) at 37 °C under 5% CO_2_. All cell lines were authenticated by short tandem repeat (STR) fingerprinting at the Medicine Laboratory of the Forensic Medicine Department of Sun Yat-Sen University (SYSU) (Guangzhou, China) and were found to be free of mycoplasma contamination. ZIKV ZG-01 strain (China, 2016, GenBank accession number KY379148) was a kind gift from Professor Xi Huang (Sun Yat-sen University, Guangzhou, China) [73].

### 4.2. Compound Preparation

The strain *Trichoderma* sp. SMU 410205 was isolated from a sponge (*Callyspongia* sp.) sample that was collected near Weizhou Island (Guangxi, China) in the Beibu Gulf of the South China Sea. This strain was stored on MB agar (malt extract 15 g, sea salt 10 g, H_2_O 1 L, PH 7.4–7.8) slants at 4 °C and deposited at the School of Traditional Chinese Medicine, Southern Medical University. The ITS sequence region of the strain SMU 410205 was amplified by PCR, and rDNA sequencing showed that it shared significant homology to that of *Trichoderma*. The rDNA sequence has a 100% sequence identity to that of *Trichoderma lixii*, so it was designated as *Trichoderma* sp. SMU 410205. The culture broth was extracted with ethyl acetate and then concentrated. The extract was chromatographed to obtain eight fractions (Frs. 1~9). Using HPLC analysis, Frs. 3 was purified again to obtain ten subfractions (Frs. 3-1~3-10). Frs. 3-6 was purified by semipreparative HPLC to obtain a pure compound, which was identified as Harzianopyridone (HAR) as reported [74]. HAR, determined to have ≥95% purity by analytical HPLC, was stored at −20 °C until use and dissolved in DMSO to a stock concentration of 10 mM.

### 4.3. Cell Viability Assay

Cell viability was detected by the 3-(4,5-dimethyl-2-thiazolyl)-2,5-diphenyl-2*H*-tetrazolium bromide (MTT) assay, according to a previously described method [40]. Briefly, cells were seeded in 96-well plates at a density of 8 × 10^3^ cells per well and cultured at 37 °C for 24 h. The cells were treated with continuously diluted HAR for 48 h. Then, 5 mg/mL of MTT solution was added to the cells and incubated for 4 h before detection. The insoluble formazan was dissolved in 160 mL of DMSO and read on 96-well plates at 490 nm with an enzyme standard instrument. The assay was performed in triplicate in three independent experiments.

### 4.4. RNA Extraction, Reverse Transcription, and Real-Time PCR

RNA from cell supernatants was prepared with the RaPure Viral RNA/DNA Kit (Vazyme, Nanjing, China) according to the manufacturer’s instructions. The first-strand cDNA was synthesized using random hexamer primers. Real-time PCR was conducted using AceQ qPCR SYBR Green Master Mix (Vazyme, Nanjing, China). The viral RNA copy numbers were determined as described previously [75]. Briefly, a standard curve was generated using in vitro-transcribed viral RNA standards, and the threshold cycle (Ct) value of each sample was compared with the standard curve to obtain the RNA copy number. Experiments were performed at least three times, with triplicate replicates. The primer sets used for real-time PCR were as follows:

qp-ZIKV-primer-F: TGGAGATGAGTACATGTATG

qp-ZIKV-primer-R: GGTAGATGTTGTCAAGAAG

### 4.5. Plaque-Forming Assay

Plaque assays were performed to assess viral titers, as previously described [52]. Vero cells were seeded in a 12-well plate at a density of 8 × 10^4^ cells per well and cultured at 37 °C in 5% CO_2_ overnight. Then, the medium was removed, and 5-fold serial diluents of virus were used to inoculate the cells. After 2 h, the supernatant was removed, and the cells were overlaid with a solution of 2.4% methylcellulose in MEM supplemented and incubated for 7 days at 37 °C. Next, the cells were fixed with 4% paraformaldehyde for 30 min, washed, and stained with 2% crystal violet for 30 min. DMSO was used as a solvent control. Experiments were performed in triplicate.

### 4.6. Western Blotting Analysis

Protein expression was assessed using Western blotting, as previously reported [75]. The proteins from the cells were prepared with RIPA lysis buffer (Millipore, Bedford, MA, USA). Protein concentrations were measured with a bicinchoninic acid (BCA) protein assay (Thermo Fisher Scientific, Rockford, IL, USA). Protein samples were separated by sodium dodecyl sulfate-polyacrylamide gel electrophoresis (SDS-PAGE) and transferred onto a polyvinylidene difluoride (PVDF) membrane. Non-specific antibody binding sites were blocked with 5% non-fat milk in Tris-buffered saline (TBS) (20 mM Tris-HCl [pH 7.6], 135 mM NaCl, and 0.1% Tween 20) for 1 h at room temperature and then incubated with the following primary antibodies: anti-ZIKV E (GTX133314, 1:2000, GeneTex Inc., Irvine, CA, USA), anti-ZIKV NS5 (GTX133312, 1:1000, GeneTex Inc., Irvine, CA, USA), and anti-GAPDH (60004-1-IG, 1:5000, Proteintech, Rosemont, IL, USA). Membranes were incubated with horseradish peroxidase-conjugated secondary antibody for 1 h at room temperature, and signals were detected by enhanced chemiluminescence using a commercial kit (Thermo Fisher Scientific) according to the manufacturer’s suggested protocols.

### 4.7. Immunofluorescence

Cells were fixed in 4% paraformaldehyde for 20 min and then permeabilized with 0.2% Triton X-100 and blocked with 5% BSA in PBS for 1 h. Then, cells were incubated with an anti-ZIKV E antibody (1:500, BioFront Technologies, Tallahassee, FL, USA) for 16 h at 4 °C. After three washes with PBS containing 0.1% Tween 20 (PBST), a secondary antibody Alexa 555-conjugated goat anti-mouse IgG (Thermo Fisher Scientific, Rochester, NY, USA) was used for 1 h and then 4′,6-diamidino-2-phenylindole nuclear stain (DAPI, 1:1000, Sangon Biotech, Shanghai, China) was counterstained for 10 min. The immunofluorescent images were taken with an inverted microscope (Carl Zeiss, Oberkochen, Germany).

### 4.8. ZIKV NS5 RdRp Activity Assay

ZIKV NS5 RdRp activity was measured by the ZIKV-Gluc reporter plasmid, as previously reported [76]. 293T cells were typically co-transfected in a 6-well plate (2 × 10^5^/mL cells per well) with 10 ng reporter plasmid and 2 μg ZIKV NS5 protein expression plasmid following the addition of different concentrations of HAR. After 24 h post-transfection, Gluc activity was measured. Briefly, cell supernatants were added to a white and opaque 96-well plate and injected with 60 μL of 16.7 μM coelenterazine-h (Promega, Madison, WI, USA, #S2011) per well. The luminescence was acquired for 2 s using the Infinite F500 (TECAN, Grödig, Austria).

### 4.9. BIAcore Analysis

Surface plasmon resonance (SPR) experiments were performed on a BIAcore T100 device (BIAcore Inc., Uppsala, Sweden) using CM5 sensor chips (General Electric Company, GE, Boston, MA, USA) according to the protocol provided by the manufacturer. Recombinant ZIKV NS5 protein was immobilized on a CM5 chip. Different concentrations of HAR or ribavirin were injected at a flow rate of 30 µL/min for 3 min. Subsequently, data were collected for a 3 min association followed by a 20 min dissociation. The chip was regenerated by injecting 10 µL of 15 mM NaOH for 20 s. All procedures were run in 1% DMSO PBS-P20 (GE Healthcare, Beijing, China) as a running buffer. The binding kinetics were analyzed with the software BIA-evaluation version 3.1 using a 1:1 Langmuir binding model. The K_d_ value was calculated as previously described [42]. The recombinant ZIKV NS5 protein was the same as the protein in another article [52].

### 4.10. Statistical Analysis

Statistical analysis was performed on triplicate experiments using a two-tailed Student’s *t*-test in the GraphPad Prism 9.0 software. The data were expressed as the mean ± standard deviation (SD) of triplicate experiments. *p* values were indicated by * *p* < 0.05, ** *p* < 0.01, *** *p* < 0.001, and “ns” indicates not significant.

## Figures and Tables

**Table 1:** Efficacy of HAR against ZIKV in 3 different cell lines.

Cell Line	EC_50_ (µM) ^1^	EC_90_ (µM) ^2^	CC_50_ (µM) ^3^	SI ^4^
SNB19	0.46 ± 0.13	1.82 ± 0.01	45.17 ± 2.16	98.20
Vero	1.40 ± 0.37	5.27 ± 0.26	92.31 ± 6.66	65.94
A549	2.63 ± 0.68	11.19 ± 0.22	82.87 ± 4.94	34.53

^1^ Real-time PCR assay to confirm the 50% effective concentration that reduces virus yield. ^2^ Real-time PCR assay to confirm the 90% effective concentration that reduces virus yield. ^3^ The concentrations that result in 50% morphological changes in cells were determined using a cell viability assay. ^4^ Selectivity index, SI = CC_50_/EC_50_.

## Data Availability

The data presented in this study are available on request from the corresponding author.
